# Senescence in Bacteria and Its Underlying Mechanisms

**DOI:** 10.3389/fcell.2021.668915

**Published:** 2021-06-18

**Authors:** Ulrich Karl Steiner

**Affiliations:** Evolutionary Demography Group, Institute of Biology, Freie Universität Berlin, Berlin, Germany

**Keywords:** aging, E. coli, asymmetry, inclusion bodies, protein clusters, proteomic instability, evolutionary demography, biodemography

## Abstract

Bacteria have been thought to flee senescence by dividing into two identical daughter cells, but this notion of immortality has changed over the last two decades. Asymmetry between the resulting daughter cells after binary fission is revealed in physiological function, cell growth, and survival probabilities and is expected from theoretical understanding. Since the discovery of senescence in morphologically identical but physiologically asymmetric dividing bacteria, the mechanisms of bacteria aging have been explored across levels of biological organization. Quantitative investigations are heavily biased toward *Escherichia coli* and on the role of inclusion bodies—clusters of misfolded proteins. Despite intensive efforts to date, it is not evident if and how inclusion bodies, a phenotype linked to the loss of proteostasis and one of the consequences of a chain of reactions triggered by reactive oxygen species, contribute to senescence in bacteria. Recent findings in bacteria question that inclusion bodies are only deleterious, illustrated by fitness advantages of cells holding inclusion bodies under varying environmental conditions. The contributions of other hallmarks of aging, identified for metazoans, remain elusive. For instance, genomic instability appears to be age independent, epigenetic alterations might be little age specific, and other hallmarks do not play a major role in bacteria systems. What is surprising is that, on the one hand, classical senescence patterns, such as an early exponential increase in mortality followed by late age mortality plateaus, are found, but, on the other hand, identifying mechanisms that link to these patterns is challenging. Senescence patterns are sensitive to environmental conditions and to genetic background, even within species, which suggests diverse evolutionary selective forces on senescence that go beyond generalized expectations of classical evolutionary theories of aging. Given the molecular tool kits available in bacteria, the high control of experimental conditions, the high-throughput data collection using microfluidic systems, and the ease of life cell imaging of fluorescently marked transcription, translation, and proteomic dynamics, in combination with the simple demographics of growth, division, and mortality of bacteria, make the challenges surprising. The diversity of mechanisms and patterns revealed and their environmental dependencies not only present challenges but also open exciting opportunities for the discovery and deeper understanding of aging and its mechanisms, maybe beyond bacteria and aging.

## Introduction

Studying senescence—the decline of function with age—in bacteria has been a dichotomous field, divided into population aging on the one hand and senescence at the single-cell level on the other hand. Classically, senescence in bacteria has been concerned with population aging, the decline in viability with age of lineages, strains, or populations. An early emphasis has been on populations under stressful and suboptimal conditions, such as disinfectants. Bacteria populations exposed to disinfectants decline exponentially with time in their densities or in the numbers of viable populations (Chick, 1908). Different concentrations of disinfectants, disinfectant types, strain types, or bacteria types alter the pace of the decline, but the approximate exponential decline remains conserved (Chick, 1908). These findings are interesting, because an exponential decline implies that the force of mortality, the rate at which populations go extinct or the number of bacteria in a population decreases, is constant, i.e., no senescence is found. The implied non-senescence inspired reflections on mechanisms: if a cumulative deleterious effect of the disinfectant exists, the mortality rate should increase with time; alternatively, if the disinfectant has a selective effect acting on heterogeneity among cells in resisting the disinfectant, the mortality rate should decrease (Yule, 1910). An equilibrium balancing these two effects could theoretically lead to non-senescence, but such precise balancing seems unlikely. More than 100 years later, similar questions on mechanisms of aging remain: how do aging factors accumulate and how do differential selective effects influence senescence? The questions and mechanisms of aging studied have diversified, but nine hallmarks of aging have been carved out: genomic instability, telomere attrition, epigenetic alterations, loss of proteostasis, deregulated nutrient sensing, mitochondrial dysfunction, cellular senescence, stem cell exhaustion, and altered intercellular communication. We will consider these hallmarks in the following sections in the light of bacteria aging. We discuss conflicting results and some surprising challenges we are confronted with when studying bacteria aging.

## Individual Heterogeneity and Senescence

Concepts of differential selection acting on heterogeneity among individuals and accumulating aging factors that influence physiological function and mortality, as found in the early studies on exposing bacteria to disinfectants, remain central in the study of senescence in bacteria and beyond. For instance, senescence patterns at the population level, such as late age mortality plateaus, are found in bacteria populations, but also metazoans including *Caenorhabditis elegans*, humans, and other organisms (Vaupel et al., 1998; Jones et al., 2014; Steiner et al., 2019). Such plateaus can arise out of selection acting on random accumulation of an aging factor, which generate heterogeneity in accumulated damage, or such plateaus arise out of differential selection on heterogeneity determined at birth (Vaupel and Yashin, 1985; Weitz and Fraser, 2001). Such heterogeneity determined at birth or gained throughout life has not only become of interest to basic aging research but also to applied questions of increased antibiotic resistance in healthcare. Differential selection becomes apparent for bacteria cells that can persist high doses of antibiotics. Such persistence is achieved without developing genetically fixed resistance. The current debate centers around the question whether cells are born as persister cells or switch in a stochastic or induced manner to such states during their lives, and findings suggest that these options are not mutually exclusive but differ in their frequency (Balaban et al., 2013; Brauner et al., 2017). Despite inferring on bacteria aging mechanisms from population level studies, deeper insights can be gained from single-cell investigations, because such investigations allow for comparison and heterogeneity in life courses among cells. For the remainder of this article, such single-cell senescence is our focus.

## Cell Senescence in Bacteria

**FIGURE 1:**
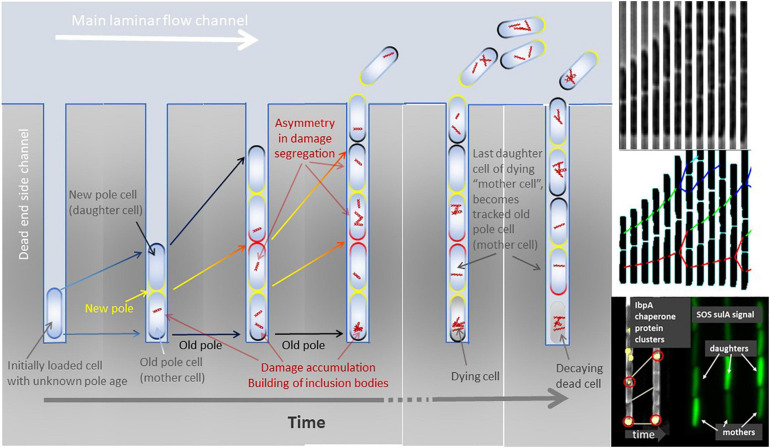
**Left panel:** Cartoon of a mother machine channel across time. The initial loaded cell (left most channel in the panel) is of unknown cell wall age and polarity. After its first division, a new cell wall is built at the mid cell planar plane and becomes the new pole (yellow). The opposite cell poles define the old poles (dark). Pole age increases with time and division (yellow new pole, red poles built at previous division, black poles built at least two divisions ago). Over multiple divisions, the cell with the oldest pole remains at the bottom of the dead-end side channel and can be tracked until it dies, decays, and finally dissolves. Other cells than the bottom-most cell are pushed out of the side channels and washed away by the laminar flow. Damage, illustrated by red intracellular structures, accumulates throughout the life of a cell and can be purged by asymmetric division. Damage repair and recycling is not visualized here. Once the bottom-most old pole cell (mother cell) dies, its last produced daughter cell becomes the new cell that can be tracked throughout its lifespan. **Right top and middle panels:** Time sliced growth and division of bacteria cells growing in a dead-end side channel of a mother machine (phase contrast images top) and its lineage tracking with divisions after image analysis (**middle panel**). **Right bottom panel:** Examples of asymmetric division of fluorescently marked protein clusters (IbpA chaperones) and asymmetric segregation of transcription factor signal related to SOS stress response (sulA) among old pole (mother) and new pole (daughter) cells.

Historically, little interest has been given to senescence at the single-cell level in bacteria, because morphologically symmetric dividing bacteria were thought to be immortal and flee senescence (Williams, 1957; Partridge and Barton, 1993; Moger-Reischer and Lennon, 2019). The argument goes as follows: a mother cell divides into two identical daughter cells, which themselves are identical to the mother, and only asymmetric reproduction, such as a soma and a germline, would allow for senescence as postulated in the disposable soma theory of aging (Kirkwood, 1977). Over the last two decades, this notion of non-senescence and immortality in bacteria has been overhauled. Reproductive senescence has been shown for *Caulobacter crescentus*, a morphologically asymmetric dividing bacterium (Ackermann et al., 2003). Similar results were shown for morphologically symmetrical dividing *Escherichia coli* bacteria, by studying microcolonies up to the seventh cell division in Petri dishes (Stewart et al., 2005). Stewart et al. (2005) introduced the concept of an old pole cell and a new pole cell for the rod-shaped *E. coli* bacteria. The cells built at each fission at the axial center plane a new cell wall that becomes the new pole, whereas the distal, retained cell wall defines the old pole. The old pole cell wall is retained, and therefore, the age of a cell can be determined by the age of the old pole; hence, not all old pole cells have the same age (Figure 1). Analogies to mothers and daughters for old pole daughter cells and new pole daughter cells have been made, respectively (Wang et al., 2010; Steiner et al., 2019). The finding of Stewart et al. (2005) that the apparent morphologically symmetric dividing *E. coli* bacteria are functionally asymmetric has stirred much empirical and theoretical interest in bacterial senescence. Empirical studies performed in microfluidic devices, mainly so-called mother machines, challenged the finding of reproductive senescence in bacteria by showing that no reduced growth rates were observed between the 10th and up to the 200th division of a cell when measured in microfluidic devices (Wang et al., 2010). Despite the lack of reproductive senescence, that is, no reduction in growth rates was found in microfluidic devices, increased filamentation rates and chronological senescence, determined by increased mortality with age, were detected (Wang et al., 2010). Filamentation is the continued growth of a cell without division and is a stress response, indicating that stress responses might play a central role in bacteria senescence, as suggested for eucaryotic cells (Bi and Lutkenhaus, 1993). The link of aging to stress in bacteria is strengthened by the link between the rate of aging and the RpoS pathways, a general stress response (Yang et al., 2019). RpoS activity inhibits cell division under nutrient-limiting conditions, making such linkage physiologically plausible (Mauri and Klumpp, 2014).

In attempts to understand the discrepancies among studies that found or did not find reproductive senescence, i.e., a decline in growth rates with age, Rang et al. (2012) concluded that senescence in *E. coli* occurs only when an extrinsic stressor is present, a finding similar to fission yeast where senescence under stressful but not benign conditions has been observed (Coelho et al., 2013). In yeast, reproductive senescence is mainly measured as the budding rate, the division time among budding events, or the number of budded daughter cells before budding ceases. Stressful conditions—in *E. coli* studies that found reduced growth rates, i.e., reproductive senescence—could arise from fluorescence imaging techniques, including production costs of fluorescent proteins, genetic costs associated with expressing fluorescent proteins, stress incurred by exposing cells to high energy light required for exciting fluorescent proteins, or potentially toxic substances that are used to detect cell death (e.g., propidium iodide) or that prevent cell adhesion and clustering (e.g., Tween 20). Costs are paid through damage that leads to reduced growth rates—reproductive senescence—or even increased mortality—chronological senescence. Recent studies on individual *E. coli* showed that even without extrinsic stressors, reproductive senescence occurs (Łapińska et al., 2019) and thereby contrasts the findings of another recent study that did not find such reproductive senescence without extrinsic stressors (Proenca et al., 2019). These studies used different K-12 wild-type-derived strains (BW25113 and MG1655, respectively), but were similar in growth conditions both using similar microfluidic devices and grew cells in rich media: lysogeny broth. The study that did not find reproductive senescence even used low concentrations of Tween 20, which might impose some extrinsic stress. Furthermore, under extrinsic stress, reproductive senescence as well as chronological senescence has been or has not been detected (Wang et al., 2010; Vedel et al., 2016; Łapińska et al., 2019; Proenca et al., 2019; Steiner et al., 2019). Among these studies, differences exist in duration (hours to several days), culture media (minimal medium M9, or rich medium LB), fluorescence excitation (none, to short excitation 200 ms each 4 min), temperature (mainly 37°C but up to 42°C), and strain types [most on MG1655 or BW25113, Table 1, but see also Wang et al. (2010) and Jouvet et al. (2018)]. It is apparent that morphologically similar dividing bacteria, such as *E. coli*, senesce and show functional asymmetry between daughter cells after fission under most conditions; single studies suggest that optimal environments might prevent senescence, but conflicting results exist.

**TABLE 1:** Examples of single-cell studies on aging using molecular targets.

**Aim**	**Device**	**Promoter/molecular target**	**Function of target (on protein)**	**Main findings**	**Strain**	**Experimental conditions**	**References**
Heat shock-induced PA	Agar plate	Dnak (Hsp70) (disaggregation)	Refolding	PA at poles	wt K-12 *E. coli* MC4100	LB/45°C for 20 min, then 30°C	Winkler et al., 2010
Repair deficiency	Mother machine	ΔdnaK	Knockout quality and repair	Old pole reduced growth	K-12 *E. coli* wt BW25113	LB 37°C	Proenca et al., 2019
Heat shock-induced PA	Agar plate	DnaJ (disaggregation)	Refolding	PA at poles	wt K-12 *E. coli* MC4100	LB/45°C for 20 min, then 30°C	Winkler et al., 2010
Heat shock-induced PA	Agar plate	ClpB (Hsp104) (disaggregation)	Refolding, quality control	PA at poles. Old pole with PA grow slow	wt K-12 *E. coli* MC4100	LB/45°C for 20 min, then 30°C	Winkler et al., 2010
Repair deficiency	Mother machine	ΔclpB	Knockout quality and repair	Old pole reduced growth	K-12 *E. coli* wt BW25113	LB 37°C	Proenca et al., 2019
Heat shock-induced PA	Agar plate	GroEL–GroES (disaggregation)	Refolding	No relocation	wt K-12 *E. coli* MC4100	LB/45°C for 20 min, then 30°C	Winkler et al., 2010
Heat shock-induced PA	Agar plate	Lon (disaggregation)	Degradation	No relocation	wt K-12 *E. coli* MC4100	LB/45°C for 20 min, then 30°C	Winkler et al., 2010
Heat shock-induced PA	Agar plate	ClpX	Degradation	No relocation	wt K-12 *E. coli* MC4100	LB/45°C for 20 min, then 30°C	Winkler et al., 2010
Heat shock-induced PA	Agar plate	ClpP	Degradation	No relocation	wt K-12 *E. coli* MC4100	LB/45°C for 20 min, then 30°C	Winkler et al., 2010
Heat shock-induced PA	Agar plate	HslU	Degradation	No relocation	wt K-12 *E. coli* MC4100	LB/45°C for 20 min, then 30°C	Winkler et al., 2010
Spontaneous PA	Agar plates	IbpA (sHsp)	Sequestration	PA at old pole. Old pole reduced growth	*E. coli* wt MG1655	LB 37°C	Lindner et al., 2008
Heat shock-induced PA	Agar plate	IbpA (sHsp)	Sequestration	Reduced growth independent of PA. PA increased stress tolerance	*E. coli* wt MG1655	LB/47°C for 15 min, then 37°C	Govers et al., 2018
Localizing PA	Mother machine	IbpA	Sequestration	PA located at old poles; reduced growth	K-12 *E. coli* wt MG1655	LB 37°C	Proenca et al., 2019
Glucose accumulation	Mother machine	2-NBDG, (ThT) staining amyloid aggregates	Glucose uptake	Old pole grows slow; slow glucose accumulation. No PA. Aging not linked to PA	*E. coli* wt BW21113	LB 37°C M9 glucose accumulation	Łapińska et al., 2019
Protein expression old and new pole	Agar plates	mut3b	General protein expression	Old daughter less protein expression. Old pole lineages higher asymmetry	*E. coli* K12 wt NCM3722	M9	Shi et al., 2020
Translation errors and mutations	Mother machine	MutL	DNA mismatch repair	Age-independent rate of mutations	*E. coli* wt MG1655 and mutH strain	LB 37°C	Robert et al., 2018

*The examples listed here are on *E. coli* strains and have a focus on protein aggregates. Many of the studies used in addition to the fluorescently labeled target also knockout strains of these molecular targets to gain a deeper mechanistic understanding. Either experiments were performed in a microfluidic device (mother machine) or colony growth was tracked on an agar plate. PA, protein aggregates; wt, wild type; LB, Luria–Bertani.*

## Asymmetry in Damage Distribution and Senescence

**FIGURE 2:**
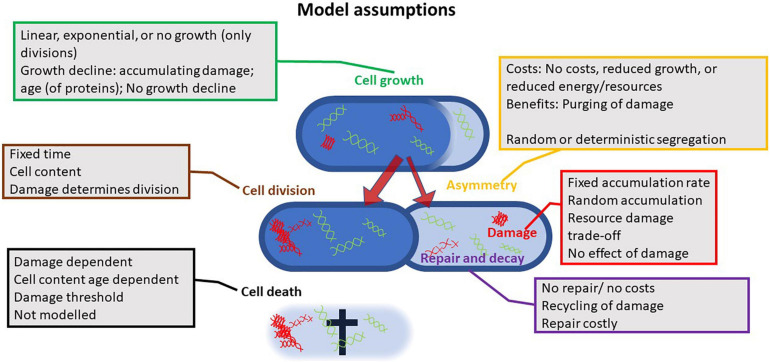
Illustration of the assumptions different models rely on; all models investigated the effects of asymmetry at cell fission. The multitude of assumptions and combinations thereof highlights how contrasting findings can be explained. Up to six parameters have been considered in such models: growth or the increase in active proteins, cell division, damage accumulation or the rate at which active proteins are transformed into passive proteins, the asymmetry at fission in damage or passive proteins, the decay of damage or the repair for passive to active proteins, and cell death. Differences in assumed cost functions, efficiencies of repair, and effect of damage further increase the diversity of outcomes.

The empirical findings on bacterial senescence were closely accompanied by mathematical models, showing how functional asymmetry turns out to be crucial for allowing partial rejuvenation of some cells to prevent population aging (Ackermann et al., 2007). Three key assumptions have been made: first, cells will accumulate damage, or any other aging factor, that reduces function with increasing age, an assumption well in line with assumed causes of aging such as oxidative processes, including DNA oxidation or other effects of reactive oxygen species (Santos et al., 2018); second, damage repair mechanisms can only slow but not prevent damage accumulation—if damage would not accumulate, cells could flee senescence (Clegg et al., 2014); and third, asymmetry in damage distribution at cell fission allows partial purging of damage in one cell by increasing damage load in the other cell (Figure 2; Ackermann et al., 2007). This third assumption of selective segregation has also been termed exogenous repair (Bell and Cambridge University Press, 1988) and is required if the first two assumptions hold. If damage would accumulate within cells (assumptions 1 and 2) and could not be purged through asymmetric divisions (assumption 3), population aging would result and populations would go extinct (Ackermann et al., 2007). High mortality and fission rates can be beneficial by purging at higher frequency damage through asymmetric division to the extent that dying cells are used as “garbage” dumps to rid large fractions of accumulated damage. Under conditions of high turnover, increased asymmetry at fission in damage load is expected, with the option of selective death of highly damaged cells (Watve et al., 2006; Evans and Steinsaltz, 2007). Contrasting these predictions, benign conditions have been associated with reduced asymmetry (Vedel et al., 2016). Considering most assumptions of these models, extreme damage segregation might not lead to maximum population growth rates, that is, perfect symmetry or complete asymmetry appears to be only favored under simplistic assumptions. Model predictions range between complete asymmetry, intermediate asymmetry, or no asymmetry (Watve et al., 2006; Ackermann et al., 2007; Evans and Steinsaltz, 2007; Erjavec et al., 2008; Chao, 2010; Rashidi et al., 2012; Clegg et al., 2014; Moger-Reischer and Lennon, 2019; Blitvić and Fernandez, 2020). This implies for populations segregating the accumulating damage in an optimal asymmetric fashion, whose lines of descendants that continuously inherit the larger fraction of damage go extinct after some variable time, while the population—consisting of many lines, including those that inherit small fractions of damage—remains viable.

Damage accumulation, damage segregation at cell fission, and damage-dependent selective mortality are balanced in a way that damage distribution stabilizes across the population level, but not within the individual cell. Within cells, damage load can be highly dynamic, dependent on individual damage aggregation and damage segregation (Weitz and Fraser, 2001; Steiner et al., 2019). As in other optimization models, findings depend on assumed costs. In bacteria, senescence cost is associated with cell growth, damage repair, damage accumulation, asymmetry, and mortality. Various models predict non-senescence, for instance, when repair rates exceed damage rates and thereby any accumulation of damage is prevented (Clegg et al., 2014). This model contrasts with the second key assumption mentioned above that repair rates cannot exceed damage rates. Other models that do not predict senescence but predict asymmetry in segregation are based on modified population genetic models (Rang et al., 2011, 2012; Proenca et al., 2018, 2019). In those models, one-cell lineage, the one corresponding to the lineage repeatedly inheriting the maternal old pole cell, attracts to one equilibrium growth state that defines lower fitness (lower cell division rates) and a second equilibrium attractor growth state with slightly higher fitness (higher cell division rates) that corresponds to the lineage that sequentially receives the maternal new pole. Rejuvenation occurs in the new pole lineage and any damage that accumulates between cell divisions is transmitted to the old pole lineage. In these old pole lineages, damage does not accumulate over multiple divisions. Cells exist between the new pole lineage and the old pole lineage attractor states, but empirical observations suggest that these cells converge to the respective attractor state within three divisions. The crux of these models is that the amount of accumulating damage between two divisions cannot exceed the dilution effect achieved through cell growth and fission; otherwise, the old pole lineage would age and go extinct (Evans and Steinsaltz, 2007). Also, under such assumptions, the growth rate (fitness) of the two types of lineages is not equal since faster dividing lineages will grow faster and therefore contribute to larger fractions to the overall population. This fitness difference due to differences in generation times results in non-stable equilibria at the population level, though at the single-cell lineage level, equilibria might be observed (Blitvić and Fernandez, 2020). The model predicting two growth equilibria is supported by empirical data under benign conditions where no senescence, with respect to cell growth, has been observed (Proenca et al., 2018, 2019), though other studies performed under conditions that mirrored largely the study conditions of Proenca et al. (2019) revealed senescence by illustrating declining growth rates (or increased doubling times) with age (Łapińska et al., 2019). Non-senescence seems a rare event and most studies detected senescence in at least some traits, but in those studies, some level of extrinsic stressors, such as increased temperature (high energy), light exposure to excite fluorescein, potentially toxic chemicals that are used to determine cell death (propidium iodide) or prevent cell agglomeration, and biofilm forming (e.g., Tween 20), cannot be ruled out (Wang et al., 2010; Winkler et al., 2010; Steiner et al., 2019). To conclude on theoretical approaches, all models motivate their assumptions based on empirical findings, and the diversity of theoretical predictions reflects on the inconsistencies of empirical results (Figure 2). Despite these inconsistencies, the models help to understand changing optima for asymmetry dependent on environmental conditions, as suggested by empirical findings. Unfortunately, senescence and asymmetry in simple organisms such as bacteria are not as simple as one might hope. Perfect rejuvenation, where cells are left without any damage, as realized for metazoans, might not be optimal for unicellular organisms (Evans and Steinsaltz, 2007; Moger-Reischer and Lennon, 2019). Even though these theoretical models rely on partly fundamental different methodological approaches, such as branching processes models, super processes models, structured matrix models, or classical differential equation models, they all agree in their prediction of asymmetry as found in all empirical studies.

## Mechanisms of Aging

The potential for revealing aging mechanisms in bacteria is considered substantial, since various bacteria species—*E. coli* as a foremost example—are model systems for molecular exploration (Stewart et al., 2005). In addition, their somewhat simpler biology suggests them as model systems to link aging phenotypes and mechanisms. Considering the nine hallmarks of aging defined for metazoans—genomic instability, telomere attrition, epigenetic alterations, loss of proteostasis, deregulated nutrient sensing, mitochondrial dysfunction, cellular senescence, stem cell exhaustion, and altered intercellular communication—bacteria, as unicellular organisms, do not require distinction between organismal senescence and cellular senescence, as various other hallmarks of aging can be excluded in bacteria (López-Otín et al., 2013). Bacteria do not have stem cells; therefore, stem cell exhaustion does not need to be considered. Most bacteria lack telomeres; therefore, telomere attrition requires no consideration. Bacteria lack mitochondria; therefore, no increased mitochondrial dysfunction with age can influence senescence—though oxidative stress-related mechanisms show similarities between metazoans and bacteria (Imlay, 2013; Janikiewicz et al., 2018).

Altered intercellular communication with increasing age, another hallmark of metazoan aging, is little explored in bacteria. In metazoans, such age-specific intercellular communication has been described in the context of chronic low-grade inflammation (López-Otín et al., 2013; Zhang et al., 2013). In a single-celled organism, intercellular communication might be expected to be less important, but that seems not to be the case. In the context of aging, at least, intercellular communication is no prerequisite for detecting senescence in bacteria. Most aging research in bacteria at the single-cell level is done using microfluidic devices, but despite preventing most intercellular communication in microfluidic chips, chronological or reproductive senescence is found (Wang et al., 2010; Łapińska et al., 2019; Steiner et al., 2019). Obviously, observing senescence under conditions that prevent intercellular communication is not sufficient to rule out the potential impact of altered communication at old age for senescence. Still, some evidence of limited influence of altered intercellular communication at old ages comes from studies on microcolonies grown on Petri dishes, where intercellular communication is allowed. Senescence patterns show similarities between microcolony studies and studies done in microfluidic devices, with the former being limited in time depth (up to 7–10 divisions) (Stewart et al., 2005; Wang et al., 2010; Winkler et al., 2010; Łapińska et al., 2019; Steiner et al., 2019). Intercellular communication is intensively explored in biofilm studies, with aging biofilms showing altered bacteria behavior for, e.g., *Bacillus subtilis* (Bartolini et al., 2019). Whether the changed behaviors of cells in aging biofilms are due to nutrient depletion, crowding, quorum sensing, or altered intercellular communication is not yet known. In senescing biofilms, cells show general stress response, like other aging phenotypes, but senescing biofilms rather mirror settings of population aging than single-cell senescence. The complexity of biofilms confronts us, in a very general way, with challenges that might suggest a simpler approach, such as controlled intercellular communication among single cells in microfluidic devices to explore potential altered intracellular communication with age in bacteria (Gupta et al., 2020).

Age-specific nutrient sensing, another hallmark of metazoan aging, is maybe most prominently known for the TOR and insulin/IGF-1 pathway in metazoans, but these pathways seem not to function in bacteria. Bacteria use for nutrient sensing various known mechanisms, but they appear to be distinct from metazoans and other organisms (Chantranupong et al., 2015; Bhattacharyya et al., 2018; Alvarado et al., 2020). Known bacteria sensing mechanisms involve for instance PII (sensing of glutamine) or various chemoreceptors, e.g., Tar, Tsr, Tap, Trg, and Aer, but their age specificity is little explored, not to mention at the single-cell level. At least, the described molecular mechanisms involved in nutrient sensing pave the way for age-specific exploration. Indications that dietary restriction can reduce mortality in *E. coli* are an encouraging sign of physiological and environmental integration for senescence, and what role altered nutrient sensing might play remains to be determined.

What remains of the metazoan hallmarks of aging for bacteria are genomic instability, loss of proteostasis, and epigenetic alterations. These three hallmarks belong, with telomere attrition, to the four primary hallmarks that are stated as undeniably negative in their effects. Most work on the mechanisms of bacteria senescence has been on the loss of proteostasis, with a focus on asymmetric division of inclusion bodies, but very insightful findings on mutation rates explored at the single-cell level along the age axis exist, while epigenetic explorations are less explicitly studied.

### Genomic Instability and Senescence

Genomic instability in bacteria is substantial, foremost evidenced by the exceptional evolvability of bacteria populations (Darmon and Leach, 2014). Despite genomic instability being intensively studied in bacteria, exploring aging-related questions on genomic instability at the single-cell level is rare. Bacteria still provide exciting opportunities for exploring age-specific genomic instability. Genomic instability is generally associated with either the frequency of occurrence of DNA damage, or epigenetic or mutational reduction in expression of DNA repair genes. In short, the mutations originating from replication errors depend on both the rate at which errors occur and to what rate they can be repaired, and for senescence, whether these rates change with age. Quantifying such rates at the single-cell level in a high-throughput manner has only recently become feasible. Using fluorescently labeled MutL mismatch repair proteins that form fluorescent foci, replication errors can be visualized and thereby quantified in live cells at the single-cell level (Elez et al., 2010; Table 1). Comparing wild-type strains, holding a functional repair system, to strains with an inactive mismatch repair (MutL deficient), allows quantifying both mutation rates and repair accuracy (Robert et al., 2018). Replication errors occur at a constant rate across ages and followed a Poisson distribution. Such a pattern of Poisson distributed errors suggests that events occur independently of each other, i.e., replication errors that resulted in mutations occur at a constant rate across life and, hence, independent of age. This averaged constancy in mutation rates over age does not prevent variation within one and the next division of a cell. Replication error rate within a single-cell cycle varies by a factor of ∼3 and is associated with the number of replication forks, i.e., replication error correlates with the size of the cell (Robert et al., 2018). Slowly growing cells, which might be biologically older with more accumulated damage (accumulated mutations), did not show higher mutation rates for the remainder of their lives. We need to caution that these findings relate to mutation rates for replication errors and remind ourselves that stress-induced mutagenesis acts mainly at the level of error repair, and there might be age-related changes in repair efficiencies. Furthermore, benign environments reduce stress-induced mutagenesis, which agrees with the observation of non-reproductive senescence in these fascinating studies on mutation dynamics (Robert et al., 2018).

Comparing mutation rates for WT strains and repair-deficient mutants at ∼0.0022 and ∼0.32 mutations/h, respectively, shows that most mutations that occur during replication are efficiently repaired (1 out of 145 mutations slips the repair) (Robert et al., 2018). Such high rates of repair are not surprising given the effort bacteria invest to detect errors; *E. coli* cells produce every 10 min enough base excision repair enzyme to scan the entire chromosome (Lee and Wallace, 2017). Concerns that the application of heterologous fluorescence markers and high energy excitation light could increase mutation rates and aging can partly be relaxed, as the mutation rates quantified at the single-cell level using these methods of potential concern mirror estimates based on whole-genome sequencing (Lee et al., 2012). Mutations that slipped repair did not lead to reduced growth and no evidence of senescence with respect to growth was revealed. However, for cells with deficient repair and therefore increased number of mutations, the mean growth rate slightly decreased. These repair-deficient cells either reduced growth in a stepwise manner, suggesting that single mutations caused growth reduction or ceased growth completely and finally died (Robert et al., 2018). At the single-cell level, heavily deleterious mutations are found but are rare (∼0.3% of all mutations). Heavily deleterious mutations could hence only account for the senescence of a few cells, not enough to drive senescence patterns across cells in a population; most non-lethal mutations had small fitness effects. These precisely quantified numbers at the single-cell level, in combination with the findings that mutations occur at a constant rate and that current and future mutations are independent of the accumulation of previous mutations, strongly suggest a minor role of genomic instability for senescence in *E. coli*. Together, no evidence is revealed for increased genomic instability with age, and rare non-lethal mutations with stronger deleterious effects are not sufficient to shape observed senescence patterns at the population level, despite them being inherited to their daughter cells. In yeast cells, genomic destabilization has been shown, and this instability has been associated with mitochondrial fragmentation and dysfunction that showed age-specific patterns (Moger-Reischer and Lennon, 2019).

### Loss of Proteostasis

Mechanistic aging research in bacteria centers around loss of proteostasis, with an emphasis on studying inclusion bodies. Inclusion bodies consist primarily of aggregated damaged and misfolded proteins; for additional details on inclusion bodies, we refer to an excellent recent review (Schramm et al., 2019). Inclusion bodies, i.e., aggregates, appear in response to metabolic activity associated with cell growth and extrinsic or intrinsic stresses, including antibiotics, temperature, osmolarity, ionic strength, pH, heavy metals, hypochlorous acids, and macromolecular crowding. The depletion of intracellular ATP seems also be linked to the emergence and disappearance of aggregates (Pu et al., 2019). Important for senescence, aggregates increase in number and size with cell age (Maisonneuve et al., 2008). Emerging small aggregates cluster to larger aggregates that vary in size; heat shock-induced aggregates are estimated to be composed of 2,400–16,500 protein molecules, and approximately 1.5–3% of all proteins in the cytosol are found in such aggregates after heat shock (Winkler et al., 2010). Aggregate emergence, growth, and size can be visualized using fluorescently labeled chaperones involved in quality control, degrading, refolding, and recycling misfolded or damaged proteins. Chaperones include, e.g., DnaK, DnaJ, GrpE, GroEL–GroES, ClpB, ClpX, ClpP, HslU, IbpA, and IbpB to name a few (Table 1), and several of these chaperones are also found in metazoans, including humans (Hartl and Hayer-Hartl, 2002). At emergence, aggregates are distributed relatively randomly within the cell, but then move toward the old pole of the cell over multiple-cell fission events (Lindner et al., 2008; Proenca et al., 2019). Contrasting to yeast and cells of metazoans, where within the cell, movement of inclusion bodies is energetically costly (Nyström, 2005), in bacteria, this movement is a passive—non-energy requiring—process potentially driven by crowding of DNA in the center of the cell where the new cell poles are built (Winkler et al., 2010; Coquel et al., 2013, but also see Rokney et al., 2009). Note that aggregation is non-energy demanding, while disaggregation, i.e., the chaperone activity itself, is energy demanding (Winkler et al., 2010, again see Rokney et al., 2009 for conflicting results).

Inclusion bodies became a focus of mechanistic aging research in bacteria for their assumed universal deleterious effects and them being one of the end products in a chain of reactions triggered by reactive oxygen species (Sabate et al., 2010; Moger-Reischer and Lennon, 2019). Interest increased when it was shown that inclusion bodies segregated asymmetrically with inclusion bodies being frequently located at the old pole of the cell after a few divisions and the discovery of correlated reduced growth of old poled cells (Lindner et al., 2008; Winkler et al., 2010). Beyond old pole cells showing reduced growth and division rates with increasing age, the amount of misfolded proteins correlates with the number of dead cells in bulk populations (Maisonneuve et al., 2008). Detailed analysis revealed that new pole cells that lost polar aggregates through asymmetric division grew significantly faster than new pole cells that still held aggregates (Winkler et al., 2010). Recently, more and more mechanistic understanding is gained, for instance, under benign conditions where no senescence is observed, an equilibrium in asymmetry and cell elongation can be found in repair mutants that lack specific repair chaperones (ΔclpB, ΔdnaK), but when ΔdnaK is knocked out, new daughter lineages remain at their equilibrium, while old daughter lineages show increased mortality (Proenca et al., 2019). Therefore, an intact repair capacity is not conditional for observing non-senescence with respect to growth and division rates. Growth differences among old pole and new pole cells also correlate with differences in physiology, with old pole cells accumulating glucose at a slower rate (Łapińska et al., 2019). Lower protein expression is observed in older daughter cells compared with young daughters, and old daughters produced daughters that were more asymmetric in protein expression (40%) compared with young daughters (10%) (Shi et al., 2020). Also, strains possessing the ability to create minicells from the pole regions, and thereby ejecting aggregates, grew faster after such ejection (Rang et al., 2018). All these findings support that protein aggregates contribute to physiological heterogeneity among cells (Mortier et al., 2019). Despite these encouraging findings, to date, an unambiguous causal relationship between senescence and inclusion bodies has not been established. A causal relationship of inclusion bodies and senescence is rather questioned, including a lack of correlation between cell growth rate and fluorescent concentration for cells holding aggregates (Govers et al., 2018).

Under benign conditions, no or few inclusion bodies occur (Łapińska et al., 2019). The prevention of building observable protein clusters supports the assumption that misfolded proteins can be immediately repaired or recycled under certain conditions (Clegg et al., 2014), but the notion that no senescence has to be observed (Rang et al., 2012) does not hold. Even under benign conditions where no inclusion bodies could be detected, senescence was still observed (Łapińska et al., 2019). Therefore, stressful conditions or inclusion bodies are not a prerequisite of senescence. Furthermore, inclusion bodies might not be universally deleterious in their effect, but rather a stress response induced by extrinsic factors including experimental conditions such as exposure to high energy light, heat shock, peroxide, and antibiotics (Govers et al., 2018). When exposed to heat shocks, cells with inclusion bodies showed higher adaptive potential and fitness to further heat shocks, exposure to antibiotics, or reactive oxygen species (Govers et al., 2018). This fitness advantage might be gained by inclusion bodies providing a reservoir to buffer negative fitness effects of stochastic environments (Baig et al., 2014b). The argument of inclusion bodies providing a reservoir to buffer severe conditions is supported by observations of a small number of inclusion bodies under stressful conditions and the decomposition of inclusion bodies to the extreme of complete disappearance (Baig et al., 2014a; Govers et al., 2018). More evidence against strict deleterious effects of inclusion bodies comes from studies on the interaction between DNA damage and mistranslation of proteins. Mistranslation enhances survival under changing and stressful conditions, shown by high mistranslation increasing the phenotypic tolerance and genetical resistance under DNA damage, as well as survival under temperature stress. Decreasing the basal mistranslation rate, however, reduces cell survival (Samhita et al., 2020). Such findings strengthen the interconnectedness among damage at the DNA level and damage at the protein level, both processes that have been argued to be the central mechanisms of aging.

The pace at which inclusion bodies can be decomposed also depends on the type of agglomerates as well as on the degree to which inclusion bodies are harmful. Agglomerates that contain many proteins for which misfolding has been triggered by heat shock or cells entering stationary growth phase (as in the experiments by Govers et al. (2018), described above) are easier decomposed than agglomerates being triggered by reactive oxygen species (ROS) and reactive chlorine species (RCS). These latter processes damage proteins in a covalent way and thereby make the proteins irreversibly misfolded (Schramm et al., 2019). When inclusion bodies consist of, and are induced by, overexpression of proteins (e.g., amyloidogenic CsgA protein; Marcoleta et al., 2019), these inclusion bodies are mostly cordial to the host cell, and a fraction of the overexpressed proteins can maintain their activity in the aggregated state (De Marco et al., 2019). Such differences in decomposition properties and harmfulness can help to understand what role inclusion bodies play for senescence—to date, differentiation among inclusion body types has rarely been considered in aging studies, and the potential positive effects of the types of inclusion bodies are little explored. This differentiation might allow for generalization across taxa, since prokaryotic cells differ in the biochemical makeup of damage triggered by ROS compared with eukaryotic cells, where ROS-induced senescence processes are characterized by imbalanced ROS production and ROS neutralization (Ksiazek, 2010). To date, the linkage between inclusion bodies, protein aggregates, and cellular senescence remains elusive and questioned with various mortality pathways [e.g., *mazEF* triggered cell death in *E. coli* or *skf* and *sdp* operons in *Bacillus* (Erental et al., 2012)] that are independent of asymmetric segregation (Baig et al., 2014b).

Various studies argue that inclusion bodies are attached and anchored to the old pole cell wall after the agglomerate has moved to this pole (Proenca et al., 2019; Shi et al., 2020). Such findings contrast with substantial stochastic influences in segregating inclusion bodies when cells are only tracked over a few divisions (Winkler et al., 2010; Ni et al., 2012). Attachment to the cell wall of inclusion bodies, damage, or any other aging factors that influence mortality would also be difficult to align with findings comparing new pole daughter cells that arise from old mother cells and those that arise from young mothers. Despite the two types of new pole cells having exactly the same pole age, new pole daughter cells arising from old mothers are born at an older biological age and differ substantially in senescence compared with new born daughters arising from young mothers (Steiner et al., 2019). Therefore, the age of the cell wall itself is not determining the lifespan or growth rate of a cell, but the inherited cytosol content should (Steiner et al., 2019). The difference in senescence among the two types of young daughter cells also suggests that aging factors accumulate over time, and on average, larger fractions of this aging factor are transmitted to daughter cells. Such transmission should not lead to two growth equilibria among old and young daughter lineages, as suggested by Rang et al. (2011), but rather to the distribution of cells with different aging factors. Aging factors generally tend to increase with age, and selection is assumed to be dependent on accumulated aging factor among cells. Theoretical understanding and empirical findings on senescence patterns suggest that both the accumulation of the aging factor within the cell and the asymmetric segregation at cell fission are determined by stochastic processes (Steiner et al., 2019). Young daughters of older mothers have higher mortality rates from birth onwards compared with young daughter cells of young mothers (Lansing effect), but the lifespan of young daughters is uncorrelated to the lifespan of their young or old mothers. If mother cells increase their aging factor with age and would inherit a defined fraction of this aging factor to their daughters, one would expect a correlation in lifespan among mother and daughter cells, a pattern not found (Steiner et al., 2019). Also, if mothers would differ in their rate of accumulation and inherit a low accumulation rate, a correlation in lifespan would be expected, again, a pattern not found. Taken together, we conclude that aging factors are likely not anchored to the cell wall; in average, they accumulate with age, but this accumulation differs among individuals, and asymmetric segregation at cell fission among the old pole and new pole cell holds a large stochastic component.

### Epigenetic Alterations

Non-genetically determined heterogeneity in mortality among cells has been shown when cells have been exposed to normally bactericidal concentrations of antibiotics (Brauner et al., 2017). The non-genetic heterogeneity in phenotypes, including gene expression, growth rate, and mortality, can be increased by exposing cells to sublethal levels of antibiotics (Ni et al., 2012). More importantly, for aging and epigenetics, these characteristics can be traced back to asymmetric division events of cell lineages occurring much before the exposure to the stressor (Ni et al., 2012). This predisposition effect might be enhanced by positive feedback loops. Cells that show, prior to stress exposure, higher RpoH transcription activity—an indicator of higher investment in maintenance—are more likely to divide into cells exhibiting higher stress response and increased mortality, but a reliable link to senescence through, for instance, inclusion bodies has not been established (Ni et al., 2012). Cells inheriting the ancestral protein aggregate were more stress resistant compared with sister cells that did not inherit any protein aggregate, which could provide leads to age-related patterns of protein clustering, but such relationship could not yet be established (Govers et al., 2018). One challenge for establishing relationships among senescence- and epigenetic age-related alterations is the substantial stochastic variation that overrides such signal (Ni et al., 2012; Steiner et al., 2019). To our knowledge, no clear age-specific epigenetic alterations have yet been shown, but efforts and tailored studies seem to be lacking; future discoveries will provide a more reliable answer to this question.

## Environmental Dependencies of Senescence

The demographic rates and the functional traits senescence is mainly quantified with are environmental dependent. In consequence of this environmental sensitivity of the functional traits, senescence itself is environmental dependent. Light excitation, for instance, can influence senescence patterns of growth rates, survival rates, numbers and growth of inclusion bodies, filamentation rates, and many other physiological and biochemical characteristics associated with senescence (Wang et al., 2010; Winkler et al., 2010; Łapińska et al., 2019; Proenca et al., 2019; Steiner et al., 2019). High caloric environments trigger higher cell division asymmetry and increased frequency of senescent cells (Baig et al., 2014a). Such environmental sensitivity challenges us in identifying general senescence patterns and aging mechanisms, but at the same time opens opportunities for comparative exploration of mechanisms. Evidence for decoupling reproductive senescence—reduced cell growth and division rate with age—and chronological senescence—increased mortality rates with age—is found in various studies conducted under different media (Wang et al., 2010; Steiner et al., 2019), and though under severe starvation, a trade-off between chronological senescence and growth has been shown (Yang et al., 2019). Benign conditions can prevent senescence but such non-senescence has not been found in all studies (Proenca et al., 2018, 2019; Łapińska et al., 2019). Preliminary data on *E. coli* suggest that scaling of chronological senescence along a temperature gradient, but altered shapes of senescence patterns when nutrients—glucose availability—are changed, results similar to those found in *C. elegans* (Stroustrup et al., 2016; Jouvet and Steiner, unpublished). Assuming a role of asymmetry for senescence, not only environmental conditions allow for altering senescence by altering the level of asymmetry among old and new poled cells, but also variance in asymmetry can be changed. Stochastic variance in asymmetry is enhanced under high light conditions, while the average fraction characteristic of the asymmetry is not altered (Proenca et al., 2019). Differences in variance might not necessarily alter senescence patterns, as other studies found that asymmetric segregation of protein aggregates at cell fission was independent of nutrient conditions (Baig et al., 2014a). Somewhat contrasting, different nutrient conditions triggered different selective forces on asymmetric cell division as explored in evolution experiments (Lele et al., 2011). Asymmetry in cell division might not only be environmentally but also partly genetically controlled. Nutrient-sensitive senescence is expected as the rate of aging is linked to stress response (RpoS) pathways (Yang et al., 2019), with RpoS activity inhibiting growth and nutrient assimilation (Mauri and Klumpp, 2014). More systematic exploration of environmental impact on senescence is needed. To date, we do not even have an understanding of the influence of basic conditions such as starting experiments with stationary phase cells or exponentially growing cells (Winkler et al., 2010; Proenca et al., 2019). It is evident that environmental conditions can scale and alter the process of senescence within cells, though scaling and altering senescence is not limited to environmental conditions, as evidenced by differences among *E. coli* strains exhibiting different senescence patterns under highly controlled environmental conditions (Jouvet et al., 2018).

## Perspectives

The major challenge for aging research according to the influential review on hallmarks of aging is “to dissect the interconnectedness between the candidate hallmarks and their relative contribution to aging” (López-Otín et al., 2013). Even though the nine hallmarks have a focus on metazoan aging, they should generally be applicable and show many similarities to prokaryotic senescence, including the importance of asymmetry for rejuvenation and aging, as well as basic senescence patterns such as an exponential increase in mortality earlier in life followed by a late age mortality plateau (Moger-Reischer and Lennon, 2019; Steiner et al., 2019).

The hope, to establish bacteria as simple model systems for aging, roots in bacteria being molecularly well explored, similar to other model systems that have shed new light on aging questions, including yeast and *C. elegans* (Stewart et al., 2005; Moger-Reischer and Lennon, 2019). Despite fascinating studies and approaches, the potential for gaining a much improved mechanistic understanding of senescence—including costs and roots of repair, maintenance, and longevity—has not yet been exploited to its full extent. Many bacteria studies approaching aging mechanisms from a molecular angle miss adequate time depth and are limited to a few divisions, while other studies, approaching bacteria aging from a demographic, ecological, or evolutionary perspective, fail to exploit the potential the deep molecular knowledge bacteria systems offer. Bridging the gap between organismic aging research and molecular mechanism remains elusive, which might explain why even to date no conclusive evidence for causal mechanisms of aging exists in bacteria. Bridging the molecular and organismic approach might help to establish bacteria as a model organism for aging.

### Quantifying Mechanisms

Another challenge to dissect the interconnectedness between the candidate hallmarks of aging is a qualitative rather than quantitative evaluation of (molecular) mechanistic influences on senescence. Studying populations in bulk cultures, even when deeper molecular knowledge is applied, makes it challenging to quantitatively separate senescence at the individual and population level, similar to the challenges of dissecting population and individual senescence known from the early explorations on disinfectants (Chick, 1908; Yule, 1910). Qualitative evaluation does not dissect among mutually non-exclusive mechanisms. An example can be given by the manifold influences of ROS on senescence. ROS-triggered processes might be involved in various hallmarks of aging, including DNA oxidation and dysregulation, and loss of proteostasis where ROS might trigger inclusion bodies. Without a quantification, separating out these processes for fitness at different levels is not possible, and such quantification requires, in large, an evaluation at the individual level. There are specific conditions, such as stationary phase populations, for which it was shown that when incubated in the absence of oxygen, they show significantly extended lifespans compared with populations grown in the presence of oxygen (Dukan and Nyström, 1998). These findings link ROS and oxidative stress to stationary phase-associated senescence, but even at stationary phase, a small turnover of cells exists, making it difficult to exactly quantify contributions. On the other extreme of resource availability, under very benign conditions, even when oxygen is available and exponential growth is achieved, bacteria cells often switch to anoxic and somewhat wasteful metabolism (Basan et al., 2015), but extensions of lifespan under such benign conditions remain controversial (Baig et al., 2014a; Proenca et al., 2019). Preliminary evidence exists for calorie restriction to act in bacteria as it has been shown for yeast (Taormina and Mirisola, 2014; Yang et al., 2019). As in eukaryotes for bacteria, the positive role of ROS, e.g., as an important signaling and regulating factor and not only as a damaging agent, is not well understood (Santos et al., 2018), as well as its role for bacterial senescence. Aside of the abovementioned results on increased lifespan of stationary phase populations under anoxic conditions, reproductively arrested populations of *E. coli* show increased population resistance to external oxidative stress and high levels of oxidative defense proteins (Maiese et al., 2009; Kwak et al., 2015). At the same time, such growth-arrested *E. coli* populations displayed high levels of damaged proteins (Dukan and Nyström, 1998, 1999) and did not show well-correlated responses between respiratory activity, protein oxidation, and lifespan (Ballesteros et al., 2001). Taking these findings together, as in yeast and eukaryotes, the importance of ROS for longevity is likely multifold and not unidirectional, likely condition dependent, and far from clear, and only a thorough quantitative evaluation at the single-cell level will allow to dissect this multifold influence (Santos et al., 2018).

The challenge—through such quantification—to identify aging factors and causal mechanisms of senescence in the first place, and their interconnectedness in the second place, is heightened by the strong stochastic aspects of senescence (Steinsaltz et al., 2019). Bacteria research has been at the forefront to quantify stochastic processes, and substantial stochastic characteristics have been shown, from transcription and translation, to the demographic fate of cells (Elowitz et al., 2002; Raj and van Oudenaarden, 2008; Jouvet et al., 2018; Steiner et al., 2019; Sampaio and Dunlop, 2020). Using fluorescent labeling of transcription and translation processes at the single-cell level has proven a powerful method for quantifying stochastic processes—methods that mirror those used for aging research on bacteria. Interest on stochastic characteristics and their functional role has risen recently and is no longer simply considered noise (Steiner and Tuljapurkar, 2012; Proenca et al., 2019). Dissecting the stochastic aspects of senescence might be needed to dissect the interconnected deterministic aspects of senescence as stated as the major challenge for the hallmarks of aging (López-Otín et al., 2013). Stochastic aspects might be similarly interconnected and scaled across levels of biological organization with feedback or snowball effects leading to self-enforcing effects or stochastic buffering across levels of organization, but we are only in the infancies to understand such interactions in stochasticity. Bacteria provide the experimental conditions for such dissection through high control of the genetic background and the environment, the tracking of trait dynamics at all levels of biological organization, and the collection of high-throughput data over a short time required for quantifying stochastic aspects.

The challenges ahead can be surmised through cross-linkage between protein synthesis and DNA repair, where global mistranslation increases cell survival under stress. Linkage becomes evident through similarities between genomic instability and protein misfolding where protein aggregation and self-assembly can be observed in proteins that regulate the DNA damage response (Xie and Jarosz, 2018). Dissecting and verifying links will remain an exciting though challenging task in bacteria, which may not be as challenging as for more complex metazoan systems. Bacteria illustrate how observations over short times, suggesting deterministic aspects of senescence, can easily be overwritten by stochastic variation when longer time frames are considered (Ni et al., 2012). Sister cells show for a brief time after cell division similar phenotypes with respect to heat shock survival, but then rapidly transition to stochastic patterns, likely due to a combination of multiple stochastic cellular processes (Govers et al., 2017). Lasting effects can still sometimes be observed: mothers that divide more slowly have daughters that divide more slowly, presumably due to larger transmission of damage (Proenca et al., 2018), but longevity does not seem to be heritable, and long-lived mothers do not have predictable longer- or shorter-lived daughters (Steiner et al., 2019). When stochastic aspects override deterministic ones, evolution, or at least the pace of evolutionary processes, is altered, and therefore, understanding stochastic influences in a quantitative way is required to answer the question on why senescence evolved in the first place (Steiner and Tuljapurkar, 2012).

### Differentiate Cause and Consequence of Senescence

The molecular toolbox is well filled for bacteria, and the ability to manipulate bacteria strains will help to differentiate among what causes senescence and what is simply a consequence of senescence. One reason why bridging molecular mechanism and organismic aging research has been challenging is the interdisciplinarity and required cross talk among labs coming from different disciplines. Recent and most promising approaches combine fluorescently labeled molecular mechanisms such as chaperones, tracking their fluorescently labeled expression dynamics in thousands of single cells throughout their lives using microfluidic devices, followed by image and data analysis, and best accompanied by theoretical understanding *via* mathematical modeling. Such expertise in molecular biology, biophysics, computational biology, statistics, and mathematics is rarely combined in a single lab. Fortunately, integration of fluorescent markers is fairly standard for most molecular labs working on model bacteria systems (Datsenko and Wanner, 2000); the number of microfluidic setups such as the mother machine increases rapidly within and beyond biophysics labs (Wang et al., 2010; Kaiser et al., 2016; Steiner et al., 2019; Sampaio and Dunlop, 2020); the recently developed deep learning-based tools overcome the bottleneck for efficient and accurate image analysis (Lugagne et al., 2020; Ollion and Ollion, 2020); and the interest on theoretical explorations and deeper analysis of precise, high-throughput, and high-quality data remains high as evidenced by previous empirical studies that have sparked numerous theoretical models (Watve et al., 2006; Ackermann et al., 2007; Evans and Steinsaltz, 2007; Erjavec et al., 2008; Chao, 2010; Rashidi et al., 2012; Clegg et al., 2014; Robert et al., 2018; Moger-Reischer and Lennon, 2019; Steiner et al., 2019; Blitvić and Fernandez, 2020). Putting these pieces together requires joint efforts, cross talk, and collaborations that have not yet been widely established. Framework programs that foster establishing such interdisciplinary consortia are popular these days, but bacteria aging has not yet profited heavily of such programs.

Aside from utilizing the molecular toolbox, experimental evolution studies on bacteria open unique insights into aging mechanisms, their role for fitness, and the fundamental question of why senescence has evolved. When taking the evolutionary perspective on bacteria studies, we need to remind ourselves that bacteria studies in the lab are far from natural conditions. Benign conditions with high division rates of 20–30 min as observed in *E. coli* are attractive to collect large quantities of data in a short time and can reveal surprising results including non- (or negligible) senescence (Proenca et al., 2018, 2019). However, bacteria in the wild have 2–50 times longer doubling time than in the lab, which illustrates that bacteria in their natural settings spend most of their life under resource-limiting growth conditions (Gefen et al., 2014; Moger-Reischer and Lennon, 2019). Without such limiting conditions, we would face a Darwinian demon and the universe would be filled with *E. coli* or similar fast dividing cells in less than a week. When considering evolutionary aspects of senescence, the fitness effect of chronological senescence, at least under laboratory conditions, is negligible, because growth differences, which determine division rates and generation times (the time to reach the average number of offspring a cell produces over its lifespan), determine fitness, while longevity does not influence fitness to an important extent. Even in such a system, where mortality might be less important than reproduction, one might reveal deeply rooted evolutionary forces shaping senescence patterns, and it is surprising to find classical patterns such as mortality plateaus in bacteria. Whether these patterns are shaped by similar forces than in metazoans remains to be determined.

Beyond the molecular toolbox and the unique opportunities experimental evolution studies in bacteria offer, comparative approaches among bacteria can provide better mechanistic understanding since bacteria differ in their strategies for instance with respect to inclusion body inheritance; some inherit inclusion bodies asymmetrically, whereas others show symmetric inheritance; some show deterministic inheritance, while others show highly stochastic inheritance (Schramm et al., 2019). Most aging research in bacteria has focused on *E. coli*, but similarities in aging mechanisms to other bacteria species exist. Aggregate inheritance in *Mycobacterium smegmatis* correlates with growth and mortality, and stress levels correlate with asymmetry in distributing aggregates between daughter cells (Vaubourgeix et al., 2015). Despite some bacteria systems starting to establish themselves as model organisms for aging, overall exploration of senescence in bacteria is limited to very few species, including *B. subtilis* (Veening et al., 2008), *Mycobacterium* spp. (Aldridge et al., 2012), or *Methylobacterium extorquens* (Bergmiller and Ackermann, 2011). This list is by no means exhaustive, but still most bacteria have not been explored for senescence. The short list of bacteria species investigated has revealed unique aspects of asymmetry and senescence, illustrating the potential for discovery and inspiration on aging research beyond bacteria.

## Conclusion

Bacteria show chronological and reproductive senescence like metazoans. The detailed investigations into mechanisms showed that aging factors being contained in the cytosol and asymmetric segregation of inclusion bodies are widely observed, even though understanding the role inclusion bodies play for senescence remains ambiguous. The diversity of empirical findings, the dependencies on environmental conditions, and the genetic background highlight the complexity of senescence. Novel insights on aging might come from bacteria studies, for instance, questioning the notion that inclusion bodies have unambiguously negative effects (Baig et al., 2014a; Govers et al., 2018). The highly controlled conditions of bacteria experiments and the large quantity of data collected at the individual level revealed the strong influences of stochastic processes on aging. If similar stochastic influences would be found in more complex metazoan systems, the dissection of the interconnectedness among the hallmarks and their contributions to aging will be challenging. The exciting technical opportunities that bacteria offer, in combination with the molecular knowledge on bacteria, will provide a deeper mechanistic understanding of bacteria senescence, but these opportunities need to be explored more systematically across environmental conditions and genetic backgrounds and in a highly quantitative way at the individual level. To date, despite their large representation in the tree of life (Hug et al., 2016), only few bacteria species have been explicitly investigated for senescence (Florea, 2017). To what degree understanding of bacteria processes of aging will be insightful for metazoan aging remains to be seen. Improved survival under antibiotic exposure of cells that hold inclusion bodies might not only shed light on senescence in bacteria but might also have medical relevance for antibiotic treatment in humans. The complex influence of inclusion bodies for bacteria aging can be insightful for the development of age-related deleterious diseases in humans such as Alzheimer’s, Parkinson’s, or type II diabetes (Sabate et al., 2010), because protein aggregates correlate with the development of such deleterious diseases. Understanding age-related aggregation processes, self-enforcing feedback loops, or snowball-like-triggered deleterious effects might inspire research on such age-related deleterious diseases, but at the same time, important differences in the emergence, biochemical makeup, and decomposing of inclusion bodies among bacteria and metazoan cells exist (Mogk et al., 2018; Nillegoda et al., 2018), and such differences might even provide greater insights. Without a shift from qualitative understanding of the mechanisms involved in senescence to a quantitative dissection of their influences, we will not gain a deeper understanding of the mechanisms and the evolution of senescence. In the end, we might get held up in discussions that were well known for the classical evolutionary theories of senescence two decades ago, where the two main mutually non-exclusive theories—antagonistic pleiotropy theory and mutation accumulation theory (Medawar, 1952; Williams, 1957)—were contrasted against each other. Both antagonist pleiotropic effects and mutation accumulation likely influence senescence in bacteria, but what their respective contributions are remains elusive. Illustrating that transcriptional error rates and mutation rates are age independent (Robert et al., 2018) is one example of such qualitative investigation, though such understanding of age-independent mutation rates does not reveal a quantitative understanding of mutation accumulation. Quantitative investigation at the individual level will likely be the crux for dissecting the interconnectedness and identifying the contributions of the identified hallmarks of aging in bacteria and hopefully beyond.

## Author Contributions

US wrote and edited the review.

## Conflict of Interest

The author declares that the research was conducted in the absence of any commercial or financial relationships that could be construed as a potential conflict of interest.
